# Supporting Video-Based Psychotherapy While Asking Who Benefits and Who Remains in Treatment

**DOI:** 10.2196/113178

**Published:** 2026-10-28

**Authors:** Luciano Dias de Mattos Souza, Karen Jansen, Ricardo Azevedo da Silva

**Affiliations:** 1 Graduate Program in Health and Behavior, Universidade Católica de Pelotas, Gonçalves Chaves Street, 373, 411 C, Pelotas, Rio Grande do Sul, 96015-560, Brazil, 55 53981180444, 55 5321288404

**Keywords:** video-based psychotherapy, internet-based treatment, telehealth

## Abstract

Meyer-Keirath and colleagues found no significant difference in posttreatment symptoms between video-based and face-to-face psychotherapy. However, the wide confidence interval indicates substantial imprecision, while the prediction interval suggests that comparative effects may vary considerably across settings; neither supports a conclusion of equivalence. For health services, symptom outcomes alone tell only part of the story. Future studies should report, by randomized group, who starts treatment, who discontinues it, and who completes the final assessment. These are distinct outcomes that may differ between delivery formats and affect the interpretation of symptom results. Video-based psychotherapy is a reasonable option, but its value should be assessed across the full treatment pathway. Adequately powered noninferiority trials should identify which patients can access, remain in, and benefit from each format.

Meyer-Keirath and colleagues [1] make a valuable contribution by defining video-based psychotherapy (VBT) as synchronous videoconference psychotherapy, distinguishing it from telephone counseling, self-guided modules, and other digital interventions. Their focused comparison addresses a practical issue for clinicians and health systems deciding which delivery options to offer.

The meta-analysis synthesizes 12 randomized trials, 900 participants, and 41 effect sizes. Its findings are consistent with offering VBT as a treatment option while leaving substantial uncertainty about comparative effectiveness in particular settings. For mental health public services, the consequential question is not simply whether symptom scores differ among assessed participants, but whether each delivery pathway helps patients access, initiate, sustain, and benefit from treatment and whether patients find that care acceptable. Cultural responsiveness and treatment engagement therefore provide important dimensions for future research and implementation.

Prospective registration, duplicate review procedures, explicit eligibility criteria, structured risk-of-bias assessment, and GRADE evaluation are important review strengths. The three-level model appropriately recognizes dependence between multiple outcomes from the same trial; reporting a prediction interval makes variation across settings visible [1].

The authors properly urge caution in interpreting the main results. The pooled estimate provided a Hedges *g* of −0.09 (95% CI –0.52 to 0.33), with no statistically significant difference between modalities [1]. This is compatible with clinically consequential effects in either direction and does not establish equivalence. Equivalence requires a justified margin and uncertainty contained within it; noninferiority requires excluding an unacceptable disadvantage [2]. Even using the article’s conventional benchmark SD of 0.20, the CI extends beyond both boundaries. The authors acknowledge that nonsignificance cannot establish noninferiority and rate the certainty of evidence as low. The prediction interval (–1.65 to 1.46) further suggests that comparative effects may differ considerably across settings.

The resulting question is who benefits from each format and under which conditions, and who remains in treatment. Pooling diagnoses, psychotherapy models, outcomes, and settings addresses an important overall question but cannot establish that their comparative effects are the same across these circumstances. Of the 12 trials, 7 were conducted in the United States, 2 in Iran, 1 in Japan, 1 in Canada, and 1 in Australia [1]. Study location does not by itself establish cultural representativeness or indicate whether an intervention was culturally adapted. Nevertheless, trials by Chavooshi et al [3] and Kishimoto et al [4] illustrate how treatment and service contexts may shape interpretation.

Chavooshi et al [3] evaluated intensive short-term dynamic psychotherapy for medically unexplained pain, whereas most other trials examined cognitive behavioral approaches, and several focused on posttraumatic stress disorder. It favored face-to-face therapy and had the highest influence statistics (Cook distance 0.49; difference in betas –1.82). Excluding it changed the pooled estimate modestly (from –0.09 to 0.03) but reduced the reported between-study *I*^2^ from 65.60% to 10.89% [1]. Thus, stability of the average does not imply stability of heterogeneity. Diagnosis, treatment approach, and context differ together in this trial; the analysis cannot determine which characteristic accounts for its distinct result. Cultural adaptation is a testable dimension of implementation, rather than a post hoc explanation for the review’s heterogeneity. Evidence from psychological interventions more broadly suggests that adaptation can affect outcomes [5-7], although these studies do not establish that culturally adapted VBT outperforms face-to-face psychotherapy. Future trials could assess adaptation, acceptability, and retention alongside delivery format.

The Japanese trial by Kishimoto et al [4] enrolled patients with depression, anxiety, obsessive-compulsive, and related disorders. It established noninferiority for its prespecified primary outcome, the 36-item Short-Form Health Survey (SF-36) mental component score at 24 weeks; symptom measures were secondary outcomes [4]. The trial reported low discontinuation, and the treating psychiatrist remained responsible for each patient’s care regardless of delivery format [4]. Participants also had to be clinically stable enough to receive video treatment [4]. These features make the findings informative while limiting what can be inferred about patients beginning a new course of psychotherapy or those unable to use video care.

The review focuses on posttreatment symptoms, but a service-level comparison also requires clear denominators for treatment initiation, treatment discontinuation, and outcome assessment in each randomized group. Noninitiation, treatment discontinuation, and missing outcome assessment are distinct events and should not be conflated. If missingness depends on modality or clinical response, analyses restricted to observed outcomes may produce biased comparative estimates. Future syntheses should therefore retrieve engagement outcomes and distinguish randomized, initiating, and assessed participants. VBT may reduce logistical barriers [8] while preserving aspects of the therapeutic relationship [9], but whether these potential advantages translate into higher initiation or sustained participation should be tested directly.

American Psychological Association (APA) guidance supports tailoring evidence-based practice to sociocultural context [10]. For VBT, cultural factors should be assessed alongside privacy, connectivity, digital literacy, and patient preference. Clinicians can reasonably offer VBT as an option through shared decision-making and monitor symptoms, attendance, and safety. Health systems should avoid interpreting this review as evidence for replacing face-to-face care or assuming cost savings. Implementation decisions should also consider acceptability, feasibility, resources, and equity.

The next step is performing adequately powered, pragmatic noninferiority trials using clinically justified, diagnosis-specific margins, with intention-to-treat and per-protocol analyses. These trials should prespecify and report treatment initiation, discontinuation, and outcome ascertainment by randomized group.

This review supports expanding a carefully monitored treatment option while leaving universal equivalence unestablished. The decisive advance will be evidence showing which patients can enter, remain in, and recover through each pathway. VBT should be judged by that complete trajectory alongside its average effect on posttreatment symptoms.

## Abbreviations

APA: American Psychological Association
SF-36: 36-item Short-Form Health Survey
VBT: video-based psychotherapy
